# Hormone replacement therapy and risk of atrial fibrillation in Taiwanese menopause women: A nationwide cohort study

**DOI:** 10.1038/srep24132

**Published:** 2016-04-07

**Authors:** Wei-Chung Tsai, Yaw-Bin Haung, Hsuan-Fu Kuo, Wei-Hua Tang, Po-Chao Hsu, Ho-Ming Su, Tsung-Hsien Lin, Chih-Sheng Chu, Shih-Jie Jhuo, Kun-Tai Lee, Sheng-Hsiung Sheu, Chung-Yu Chen, Ming-Tsang Wu, Wen-Ter Lai

**Affiliations:** 1 Division of Cardiology, Department of Internal Medicine, Kaohsiung Medical University Hospital, Kaohsiung Medical University, Kaohsiung 807, Taiwan; 2 School of Pharmacy, Master Program in Clinical Pharmacy, Kaohsiung Medical University, Kaohsiung 807, Taiwan; 3 Department of Pharmacy, Kaohsiung Medical University Hospital, Kaohsiung 807, Taiwan; 4 Department of Family Medicine, Kaohsiung Medical University Hospital, Kaohsiung 807, Taiwan; 5 Department of Public Health, Kaohsiung Medical University, Kaohsiung 807, Taiwan; 6 Research Center for Environmental Medicine, Kaohsiung Medical University, Kaohsiung 807, Taiwan

## Abstract

Hormone replacement therapy (HRT) is associated with risk of vascular disease. The association between atrial fibrillation (AF), vascular events, and different HRTs, including estradiol and conjugated equine estrogens (CEE), has been controversial in previous studies. Thus, we conducted a retrospective cohort study to investigate these associations. Female patients (>45 years old) first diagnosed with menopause were enrolled from National Health Insurance Research Dataset (1998–2008). Cox regression analysis estimated risk of new-onset AF, stroke, and major adverse cardiac events (MACE) after exposure to estradiol or CEE. Of 5489 females (mean age = 55 years) enrolled, 1815 treated with estradiol and 3674 treated with CEE. Incidence per 10^3^ person-years of AF, stroke, and MACE in CEE vs estradiol patients was 2.23 vs. 0.92, 14.0 vs. 9.09, and 15.55 vs. 10.47. As compared with patients treated with estradiol, those treated with CEE had a significantly higher incidence of AF, stroke, and MACE. The adjusted hazard ratios for each category were 1.96, 1.30, and 1.26, respectively. The significant results remained similar, even after use of propensity-score-matched strategy. In conclusion, CEE was associated with a higher risk of AF, stroke, and MACE than estradiol in menopausal females. Further exploration of underlying mechanisms is necessary.

Hormone replacement therapy (HRT) is widely used to treat menopausal symptoms, but studies including the Women’s Health Initiative (WHI) have indicated that HRT is associated with an increased risk of coronary heart disease (CHD), stroke, and venous thromboembolic disease regardless of years of therapy since menopause^1^,^2^,^3^. In contrast, the results of a clinical trial showed that the risk of stroke was not significantly different between patients receiving conjugated estrogen plus progestin and those receiving a placebo^4^. Despite the uncertain effects and mechanisms of HRT on the risk of stroke, current evidence has indicated that HRT may still play a role in the incidence of stroke.

Women experiencing stroke have a higher prevalence of atrial fibrillation (AF) than men, but these gender differences remain largely unexplained^5^. In women >65 years^6^, AF is independently associated with a 22–25% increased risk of stroke and a 1.7-fold increased risk of all-cause mortality^7^. Clinically, AF is also a major risk factor contributing to ischemic stroke. However, to the best of our knowledge, there is no evidence that a higher AF incidence may result in a higher stroke incidence with HRT treatment. Furthermore, despite the importance of the relationship between AF and HRT, this relationship remains largely undescribed and controversial^8^,^9^. At present, there is still a lack of evidence regarding factors that may modulate the risks involved in HRT treatment, such as different estrogen and progestogen preparations and different doses and routes of administration. Thus, we evaluated the risk of AF, stroke, and cardiovascular diseases (CVDs) in menopausal women in Taiwan receiving different types of HRT.

## Results

### Baseline characteristics

**Figure 1:**
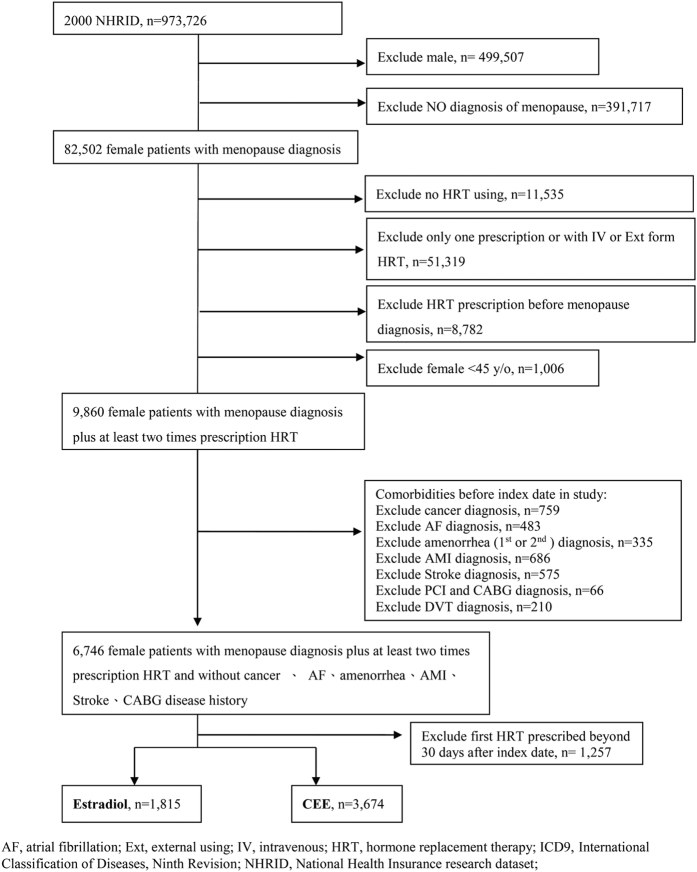
Flow chart of patient selection, with exclusion criteria.

A total of 5489 females were enrolled in the final analysis from the 2000 National Health Insurance Research dataset (NHIRD; Fig. 1). Of these patients (mean age = 55 years), 1815 were treated with estradiol and 3674 were treated with conjugated equine estrogens (CEE; Table 1). Patients in the CEE group were older and had a higher prevalence of diabetes, congestive heart failure (CHF), chronic obstructive pulmonary disease (COPD), chronic kidney disease (CKD), liver disease, and use of calcium channel blockers (CCBs) but a lower rate of sleep apnea and statin use than those in the estradiol group. The follow-up period in our patients was 7.8 [standard deviation (SD) = 3.02] years. The average drug exposure time was 0.51 (SD = 0.87) years in the estradiol group and 0.79 (SD = 1.29) years in the CEE group. The total average follow-up time in each group was 6.50 (SD = 3.04) and 8.28 (SD = 0.79) years in the estradiol and CEE groups, respectively. After matching by propensity score, we found that the baseline characteristics, including age, were comparable in two groups (Supplementary eTable 1).

### AF and stroke endpoints

**Figure 2:**
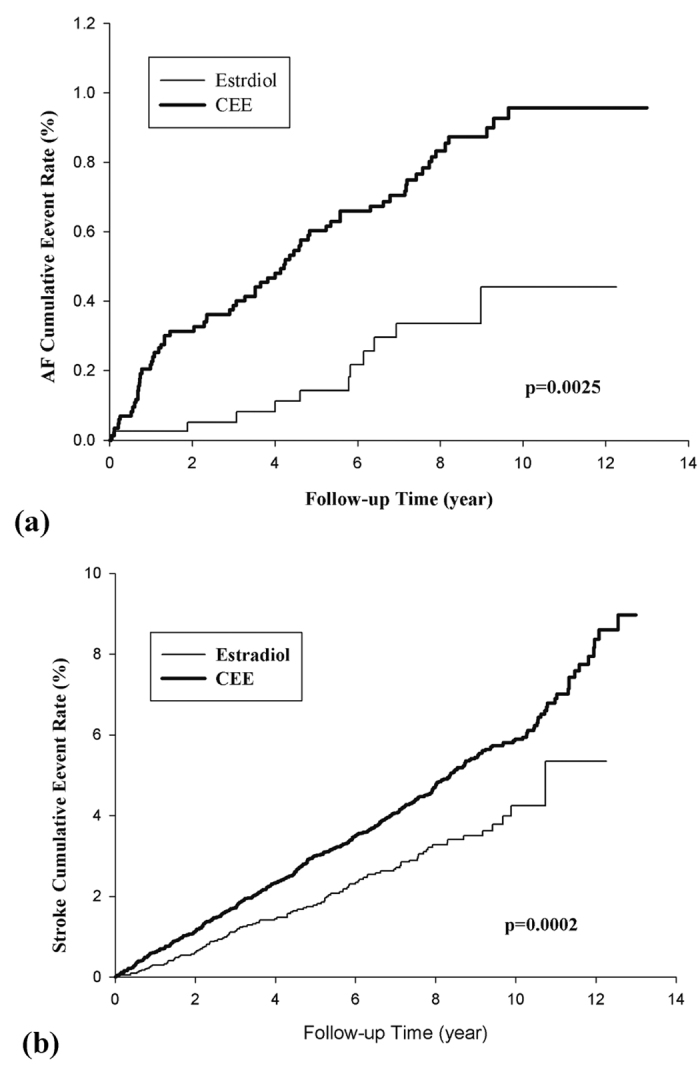
(**a**) Kaplan-Meier curves for estradiol vs CEE for AF with cumulative event rates. (**b**) Kaplan-Meier curves for estradiol vs CEE for stroke with cumulative event rates.

From a 10-year survey, 78 cases were noted with new-onset AF (11 in the estradiol and 68 in the CEE group). Furthermore, 512 subjects (105 in the estradiol and 407 in the CEE group) had a first-time stroke. The incident rate of AF was 0.92 and 2.23 per 10^3^ person-years in the estradiol and CEE groups, respectively, whereas the incident rate of stroke was 9.09 and 14.0 per 10^3^ person-years, respectively. Figure 2A,B depict the Kaplan–Meier (KM) curves for the AF and stroke incidences in the estradiol and CEE groups, respectively.

In the multivariate Cox proportional hazards models, age, valvular heart disease (VHD), and treatment with CEE were associated with AF (Table 2), whereas age, diabetes mellitus (DM), hypertension, and treatment with CEE were associated with stroke (Table 3). Other comorbidities, concomitant drug use, and income group did not associate with AF and stroke in our analysis, as shown in Tables 2 and 3. The adjusted hazard ratio (HR) for AF and stroke in patients treated with CEE versus estradiol was 1.96 [95% confidence interval (CI), 1.03–3.73, *P* = 0.042 and 1.30 (95% CI, 1.04–1.62, *P* = 0.021), respectively. The significant results remained similar, even after the matching by propensity score (Supplementary eTables 2 and 3).

### Major adverse cardiac event (MACE) endpoints

**Figure 3:**
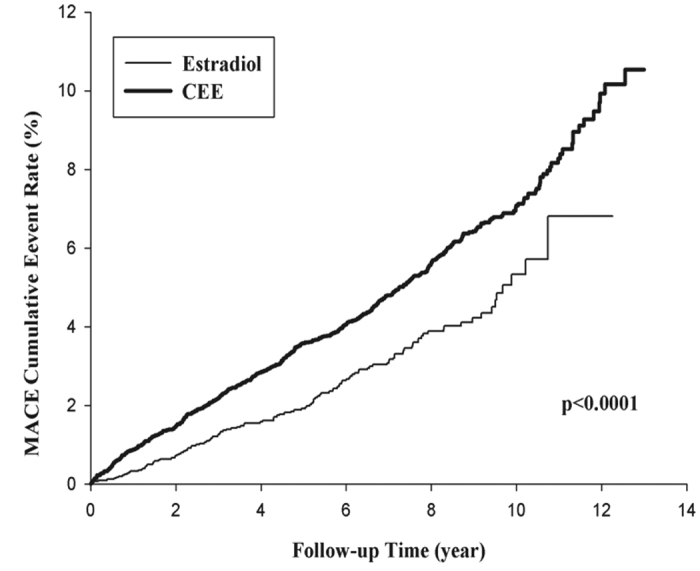
Kaplan-Meier curves for estradiol vs CEE for MACE with cumulative event rates.

In total, 606 subjects (125 in the estradiol and 481 in the CEE group) had MACEs during the follow-up period. The incidence of MACE in the CEE and estradiol groups was 15.55 and 10.47 per 10^3^ person-years, respectively. KM curves showing the difference in the incidence of MACE between the groups are presented in Fig. 3. The incidence of MACE was increased in the CEE group as compared with that in the estradiol group.

Table 4 demonstrates that age, DM, hypertension, and treatment with CEE were correlated with the incidence of MACE in a multivariate Cox proportional hazards model. The adjusted HR for MACE in the CEE group was 1.26 (95% CI, 1.03–1.54, *P* = 0.025) as compared with the estradiol group, which results were similar by the use of propensity-score-matched strategy (Supplementary eTable 4).

Regarding specific cardiac events, only the incidence of stroke was higher in the CEE group than that in the estradiol group; the adjusted HR of acute myocardial infarction (AMI) 2.23 (95% CI, 0.68–7.75, *P* = 0.179), percutaneous coronary intervention (PCI) 0.89 (95% CI, 0.43–1.85, *P* = 0.752), coronary artery bypass grafting (CABG) 1.19 (95% CI, 0.11–12.55, *P* = 0.887), and mortality 0.89 (95% CI, 0.49–1.65, *P* = 0.732) were not different between the groups, respectively.

## Discussion

In the present study, we found that CEE use in postmenopausal Taiwanese women was associated with a higher incidence of AF than estradiol use in both before and after propensity-score-matched strategy. To the best of our knowledge, this is the first study to demonstrate that CEE use is associated with a higher AF rate than estradiol use in HRT of postmenopausal women. The incidence of stroke and MACE were also higher in the CEE group than in the estradiol group in our study. The main contribution to the higher MACE incidence was a higher stroke rate in the CEE group than in the estradiol group; however, acute myocardial infarction and mortality were not increased, which is comparable with the findings of other studies^10^. The higher AF incident rate may be a causal factor for the higher stroke incidence in the CEE group than in the estradiol group.

The association between HRT and CVD is controversial. Postmenopausal hormone use could decrease the risk for MACE in women without a previous heart disease even at low doses (0.3 mg) of oral conjugated estrogen daily. However, estrogen at daily doses of 0.625 mg or greater may increase the risk for stroke^11^. The combination of CEE at 0.625 mg/d and medroxyprogesterone acetate at 2.5 mg/d significantly increased the risk of CHD, stroke, and pulmonary embolism but not all-cause mortality in WHI randomized controlled trials^2^. A secondary analysis of the same trials confirmed this trend; however, the difference was not significant, showing that HRT closer to menopause tended to reduce the risk for CHD and total mortality as compared to women more distant from menopause^3^. However, the risk of stroke was elevated regardless of years since menopause. With respect to different populations, the conclusion that HRT is associated with CVD also remains controversial^3^,^12^. In this study, we did not include the never-use HRT group for comparison due to the high possibility of non-comparable issue between the use and never-use HRT groups. Because this study was to compare the effect of different types of HRT use on the risk of AF, stroke, or MACE, rather than the comparison between the uses and never use HRT groups, we were unable to answer the relationship between HRT use and the risk of AF, stroke, or MACE.

New-onset AF was independently associated with AMI, CHF, and stroke in a group of healthy women^4^. The all-cause mortality rate was also associated with new-onset AF but not new-onset paroxysmal AF in the same study. Wolf *et al*.^6^ found that among women >75 years with CVD, those with AF were significantly more likely to be admitted with a stroke than age-matched female patients without AF even after 3 years of follow-up. There was no similar trend in men with CVD in that study. AF in women associated with stroke and mortality is an important issue. A study by Chien *et al*. demonstrated that the incidence of AF in Taiwanese women were 0.76 per 10^3^ person-years using community-based data^13^. In addition, another study also showed that the frequency of AF increased from 37 per million people 50–59 years old to 147 per million people 60–69 years old, 457 per million people 70–79 years old, and 1631 per million people 80 years old or older using the NHIRD^14^. Our data are compatible with studies that found higher AF incidence (3.15 per 10^3^ person-years or 1421 per million people) in menopausal women, particularly in the CEE group (2.23 per 10^3^ person-years) as compared to the estradiol group (0.92 per 10^3^ person-years)^13^,^14^. Similarly, our results show that stroke and MACE are also found at higher frequencies in menopausal women^15^. It may be important to use estradiol but not CEE for HRT in menopausal women to reduce the risk of AF and stroke. Furthermore, age is an important risk factor in CVD and AF^16^. Our data showed that patients in the CEE group (mean age = 55.73 years) were older than those in the estradiol group (mean age = 53.21 years). The older age of women in the CEE group may have contributed to the higher AF risk than that in the estradiol group. However, after adjusting for age in a Cox regression model with different outcomes, estradiol still showed a greater protective effect against AF, stroke, and MACE than CEE exposure.

Research regarding the association between HRT and AF is limited and controversial. Perez *et al*.^8^ found that the incidence of AF was moderately elevated in women undergoing hysterectomy and those with an intact uterus receiving CEE, but not in women with an intact uterus receiving estrogen plus progestin. Bretler *et al*.^9^ demonstrated that HRT is associated with a decreased risk of new-onset AF in female AMI patients during the first year after discharge. To the best of our knowledge, there has been no analysis of the effects of AF in different types of HRT. Instead, overall HRT was associated with a decreased risk of AF, particularly in women ≥80 years old. In our study, the cumulative incidence rate of AF was lower in the estradiol group than in the CEE group, which shows that the effects on AF in different HRT are heterogeneous.

The main biological mechanism of AF in menopausal women using HRT remains unclear. Previous data has shown that CEE has longer-lasting metabolites in humans than transdermal estradiol. Moreover, CEE and its metabolites may be a cause of inflammation, whereas estradiol has a short half-life and is not proinflammatory^17^. Inflammation has a potentially important role in mediating systemic CVD^18^,^19^. Inflammation may be a cause of hypercoagulability of the blood and a trigger mechanism for AF; therefore, CEE may increase the inflammatory effect to cause AF. Atrial remodeling and oxidative stress have been suggested to play a role in the pathogenesis of AF^20^,^21^. Xie *et al*. also identified a link between oxidative stress^22^,^23^ and aberrant intracellular Ca (2+) release via the type 2 ryanodine receptor (RyR2) that promotes AF^21^. Despite the fact that some studies have demonstrated that HRT has beneficial effects on oxidative stress in postmenopausal women^24^,^25^, studies on the direct vascular effects of HRT remain sparse, and the mechanism has not been completely elucidated. Moreover, the mechanisms linking HRT and oxidative stress or intracellular Ca (2+) to AF are not well understood. Evidence of AF in menopausal women using CEE or estradiol is currently unavailable, and further research is warranted to examine the underlying mechanism.

Several limitations were present in this study. First, because all information about drugs prescription was from claim data, we assumed that all patients completely adhered to the physicians’ instructions. However, some potential bias cannot be completely ruled out because some patients may not have used their HRT prescriptions and some may have used over-the-counter HRT. This may have resulted in some measurement error in terms of HRT exposure, including the potential systematic underestimation of HRT exposure duration, misclassification of population into cohort, or exclusion of some target populations in this study. Because those misclassifications seemed to happen in the two groups, the random misclassification, which underestimated the significance, was likely^26^. Second, the diagnosis of AF and other comorbidities from the NHIRD coding may not always be accurate. However, we have validated these diagnoses in our previous study^16^,^26^. Previous studies have used the Longitudinal Health Insurance Database (LHID) 2000 to assess the epidemiology of AF or menopause^27^,^28^. Despite the fact that there was a bias from including the number of patients using LHID 2000 in different studies, reliability was higher and standardization of the methodology was similar among different studies^27^,^28^. Furthermore, claims data can be used to identify patients with AF and menopause because of a high positive predictive value^26^,^29^. Third, no information was available regarding laboratory data, blood pressure, hormone level (in the blood), or lifestyle, which may be potential confounders. Finally, the patients in our sample were Han Chinese; therefore, generalization of the results to other ethnic or racial groups must be made with caution. In conclusion, from a large national population database, CEE had a higher risk of AF, stroke, and MACE than estradiol use for HRT in menopausal women in Taiwan. CEE was also an independent risk factor for AF, stroke, and MACE in menopausal women receiving HRT. Further exploration of the underlying mechanisms is warranted.

## Methods

### Data sources

This population-based retrospective cohort study was carried out using information from the NHIRD in Taiwan, which contains encrypted computerized outpatient care claims, hospital inpatient care, ambulatory care, dental services, and prescription drug records.

The LHID 2000 contains all the original claim data of 1,000,000 individuals randomly sampled from beneficiaries of the NHIRD in 2000, which maintains the registration data of everyone who was a beneficiary of the National Health Insurance (NHI) program during the period of 1996–2000. The NHI program enrolled about 23 million people, approximately 99% of the Taiwanese population. All registration and claim data of the 1,000,000 individuals collected by the NHI program constitute the LHID 2000. There was no significant difference in the gender distribution (χ^2^ = 1.74, df = 1, *P* = 0.187) between the patients in the LHID 2000 and the original NHIRD.

This study was approved by the Institutional Review Board of Kaohsiung Medical University Hospital (KMUH-IRB-EXEMPT-20140064). Current NHIRD and hospital regulations and guidelines did not mandate informed consent in this retrospective cohort study. All procedures performed were in accordance with the ethical standards of the institutional research committee and with the directives of the Declaration of Helsinki.

### Study sample

We identified all female patients over the age of 45 years as those having either an outpatient visit or an inpatient hospitalization with menopause, International Classification of Diseases, Ninth Revision (ICD-9: 627), diagnosed between 1998 and 2008 from LHID 2000. The date of the first menopause diagnosis was assigned as the index date.

We excluded the patients who had not been prescribed HRT after the index date or had ever been prescribed HRT before the index date. Otherwise, patients prescribed with HRT one time only were also excluded from the study population. To evaluate the systemic effect of estrogen, we included patients taking an oral form of HRT after the index date only. Patients diagnosed with stroke, cancer, AF, amenorrhea (primary and secondary diagnostic codes), AMI, deep vein thrombosis, and those receiving PCI and CABG surgery before the index date were excluded from our study population.

### Hormone replacement therapy

Female patients diagnosed with menopause who received at least two prescriptions of HRT within the follow-up time were included in this study. Patients who were prescribed estradiol after discharge were categorized as estradiol users and those prescribed with CEE were divided into a CEE users group.

To decrease the immortal-time bias because of the delay of first-time prescribed HRT after index date, we selected patients prescribed with first-time HRT within 30 days after the index date only. Additionally, patients who received a prescription of both estradiol and CEE during follow-up were excluded to avoid interactions between estradiol and CEE in our study.

### Comorbidities, prescribed medications, and study endpoints

The comorbidities of patients in our study were identified by their diagnoses in the International Classification of Diseases, Ninth Revision (ICD9) codes of one in inpatient diagnosis and one in outpatient diagnosis 1 year before the index date. The comorbidities included hypertension, DM, hyperlipidemia, liver disease, COPD, CHF, sleep apnea, thyroid disease, aortic atherosclerosis, CKD, VHD, and ischemic heart disease (excluding AMI). Income group was classified by the individual’s yearly gross income during a 1-year period before the index date. We defined the low income group according to an annual income of less than or equal to NT$894,574.00 or US$29,819.10, the national average annual household income in 2005 in Taiwan (Source: Directorate General of Budget, Accounting and Statistics, Executive Yuan, Taiwan). Alternatively, the high income group was defined as having an annual income of greater than this amount.

Data on claimed prescriptions included quantity, dispensing date, drug type, and dose. The claimed data of medication prescribed in hospital and outpatient visits were used to determine medical treatment and duration. Use of concomitant drugs was identified according to claimed prescriptions one year before the index date. These prescribed drugs included diuretics, beta-receptor antagonists, angiotensin-converting enzyme inhibitors (ACEI), angiotensin receptor blockers (ARB), calcium channel blockers (CCB), alpha-receptor antagonists, nitrates, aspirin, amiodarone, warfarin, 3-hydroxy-3-methylglutaryl-coenzyme A reductase inhibitors, Cox-II inhibitors, non-steroidal anti-inflammatory drugs (NSAIDs), steroids, and oral anti-diabetic drugs.

The endpoints in this study included first-time diagnosis of AF and MACE. MACE was defined as either stroke, AMI, PCI, CABG, or death. These endpoints were obtained by ICD-9 code in once hospital records or over twice diagnoses in outpatient claims. Information about mortality was also coded in hospital claims. Patients in this study were followed until a diagnosis of outcome or events were recorded, death occurred, or patients withdrew from the NHI. The collection period ended on 31 December, 2009. These endpoints were estimated separately over time and compared with estradiol and CEE exposure in our survey. The methods of determining the diagnostic code, protocols regarding medication administration, and the diagnosis procedures related to complications utilized in this study were validated in our previous study^16^.

### Statistics

All data are expressed as frequency (percentage) and mean ± SD. Continuous and categorical variables were compared between estradiol users and CEE users with Student’s t-test or the chi-square test, as appropriate.

Because the main interest of this study was HRT exposure and the risk of AF, each female participant was followed to accumulate person-time beginning from the index date to the newly-onset AF during an 11-year follow-up period. If patients were present to have other endpoints (stroke or MACE) or died from other causes before the onset of AF, they were censored to account for the competing risks attributable to other causes^30^. All cases with no endpoint or death occurring during follow-up were also censored.

Kaplan–Meier analysis and log-rank testing were used to examine the relationship of the cumulative incidence rate of outcomes between estradiol and CEE users. A Cox proportional hazards model was used to estimate HR of cardiovascular outcomes with estradiol and CEE after adjusting for other covariates. The covariates in the model included age, comorbidities, and administration of medications.

To consider for the issue of potential non-comparable baseline characteristics between two groups of estradiol and CEE, we also used propensity-score-matched strategy to match the covariates, listed in Table 1, as 1:2 by using a ‘greedy’ matching algorithm, with a maximum calliper of 0.1, for analysis^31^,^32^. All statistical analysis were carried out using SAS software (version 9.3; SAS Institute, Inc., Cary, NC, USA). Statistical significance was inferred at a two-sided *P*-value of <0.05.

## Additional Information

**How to cite this article**: Tsai, W.-C. *et al*. Hormone replacement therapy and risk of atrial fibrillation in Taiwanese menopause women: A nationwide cohort study. *Sci. Rep.* **6**, 24132; doi: 10.1038/srep24132 (2016).

## Supplementary Material

Supplementary Information

## Figures and Tables

**Table 1:** Clinical summary between Estradiol and CEE group.

Variable	Total (n = 5489)		Estradiol (n = 1815)		CEE (n = 3674)		P
	No	%	No	%	No	%	
Age (year ± SD)	54.90 (±7.75)		53.21 (±7.05)		55.73 (±7.94)		<0.0001
Income^a^							
High	1136	20.68	327	18.02	809	22.00	0.254
Low	4354	79.32	1488	81.98	2866	78.00	
Duration							
Total follow up (year ± SD)	7.80 (±3.02)		6.50 (±3.04)		8.28 (±2.86)		0.285
Drug exposure (year ± SD)	0.74 (±1.18)		0.51 (±0.87)		0.79 (±1.29)		0.107
Co-morbidities							
Diabetes	248	4.52	55	3.09	193	5.53	<0.0001
Hypertension	1131	20.60	357	19.67	774	21.07	0.076
CHF	84	1.53	14	0.77	70	1.91	0.001
MI other	244	4.45	94	5.18	150	4.08	0.108
Aortic atherosclerosis	26	0.47	9	0.50	17	0.46	0.928
COPD	504	9.18	147	8.10	357	9.72	0.021
CKD	111	2.02	20	1.10	91	2.48	0.001
Thyroid disease	207	3.77	69	3.80	138	3.76	0.891
VHD	40	0.73	12	0.66	28	0.76	0.612
Liver disease	489	8.91	142	7.82	347	9.44	0.020
Sleep apenea	293	5.34	128	7.05	165	4.49	0.001
Prescribed Drugs							
ACEI	390	7.11	135	7.44	255	6.94	0.716
ARB	114	2.08	41	2.26	73	1.99	0.617
BB	440	8.02	165	9.09	275	7.49	0.085
CCB	142	2.59	36	1.98	106	2.89	0.031
Diuretics	447	8.14	159	8.76	288	7.84	0.402
Nitrate	116	2.11	37	2.04	79	2.15	0.665
Statin	160	2.91	70	3.86	90	2.45	0.007
Aspirin	176	3.21	60	3.31	116	3.16	0.929
Warfarin	7	0.13	1	0.06	6	0.16	0.275
Steroid	1606	29.26	560	30.85	1046	28.47	0.267
NSAIDs	3412	62.16	1174	64.68	2238	60.91	0.189
Cox-II inhibitors	49	0.89	19	1.05	30	0.82	0.457
OAD	329	5.99	100	5.51	229	6.23	0.181
Alpha-blocker	54	0.98	18	0.99	36	0.98	0.945
Amiodarone	2	0.04	0	–	2	0.05	0.312

ACEI, angiotensin-converting enzyme inhibitors; ARB, angiotensin receptor blockers; BB, beta-blockers; CCB, calcium channel blockers; CHF, congestive heart failure; CKD, chronic kidney disease; COPD, chronic obstructive pulmonary disease; MI, myocardial infarction; OAD, oral Anti-diabetic agent; VHD, valvular heart disease; NSAIDs, non-steroidal anti-inflammatory drugs.

^a^Individual yearly gross income over NT$894,574 defined as high. The national average of annual household income in 2005 was around NT$894,574. (Source: Directorate General of Budget, Accounting and Statistics, Executive Yuan. Report on the Survey of Family Income and Expenditure in Taiwan Area of Republic.

**Table 2:** Cox proportional hazards model analysis for AF.

Variable	CHR	lower 95%CI	upper 95%CI	P-value	AHR	lower 95%CI	upper 95%CI	P-value
Age	1.10	1.08	1.13	<0.001	1.09	1.06	1.12	<0.001
DM	1.91	0.88	4.14	0.103	1.78	0.62	5.09	0.282
hypertension	3.16	2.03	4.93	<0.001	1.67	0.96	2.91	0.071
CHF	5.22	2.27	11.99	<0.001	1.89	0.76	4.69	0.169
MI	2.95	1.47	5.91	0.002	0.81	0.32	2.05	0.653
Aortic atherosclerosis	*–*	*–*	*–*	*–*	*–*	*–*	*–*	*–*
COPD	1.85	1.02	3.36	0.043	1.07	0.56	2.06	0.832
CKD	2.95	1.19	7.30	0.019	1.90	0.74	4.87	0.182
Thyroid disease	1.75	0.71	4.33	0.227	1.84	0.72	4.67	0.202
VHD	6.41	2.02	20.32	0.002	4.10	1.23	13.65	0.022
Liver disase	0.52	0.19	1.43	0.209	0.35	0.13	0.98	0.044
Sleep apenea	1.44	0.58	3.55	0.435	0.82	0.31	2.152	0.691
Treatment group								
Estradiol	1.00				1.00			
CEE	**2.58**	**1.36**	**4.88**	**0.004**	**1.96**	**1.03**	**3.73**	**0.042**

AF, atrial fibrillation; AHR, adjusted hazards ration; CEE, conjugated equine estrogens; CHR, crude hazards ratio; CHF, congestive heart failure; CI, confidence interval; CKD, chronic kidney diease; COPD, chronic obstructive pulmonary disease; DM, diabetes mellitus; MI, myocardial infarction; VHD, valvular heart disease.

**Table 3:** Cox proportional hazards model analysis for stroke.

Variable	CHR	lower 95%CI	upper 95%CI	P-value	AHR	lower 95%CI	upper 95%CI	P-value
Age	1.07	1.06	1.08	<0.001	1.06	1.05	1.07	<0.001
DM	2.62	2.00	3.45	<0.001	1.60	1.10	2.35	0.015
HTN	2.19	1.82	2.62	<0.001	1.46	1.16	1.85	0.002
CHF	1.67	0.96	2.90	0.068	0.81	0.45	1.46	0.484
MI other	2.13	1.55	2.93	<0.001	1.46	0.99	2.13	0.051
Aortic atherosclerosis	1.46	0.47	4.53	0.515	0.88	0.28	2.77	0.820
COPD	1.59	1.24	2.04	<0.001	1.23	0.96	1.59	0.107
CKD	0.97	0.55	1.72	0.916	0.70	0.3	1.26	0.235
Thyroid disease	0.87	0.54	1.41	0.578	0.79	0.49	1.29	0.351
VHD	2.08	0.93	4.65	0.074	1.54	0.63	3.77	0.343
Liver disase	1.17	0.88	1.55	0.281	0.93	0.69	1.24	0.613
Sleep apenea	0.99	0.64	1.54	0.988	0.73	0.47	1.15	0.179
Treatment group								
Estradiol	1.00				1.00			
CEE	**1.50**	**1.21**	**1.86**	**<0.001**	**1.30**	**1.04**	**1.62**	**0.021**

AHR, adjusted hazards ration; CEE, conjugated equine estrogens; CHR, crude hazards ratio; CHF, congestive heart failure; CI, confidence interval; CKD, chronic kidney diease; COPD, chronic obstructive pulmonary disease; DM, diabetes mellitus; MI, myocardial infarction; VHD, valvular heart disease.

**Table 4:** Cox proportional hazards model analysis for MACE.

Variable	CHR	lower 95%CI	upper 95%CI	P-value	AHR	lower 95%CI	upper 95%CI	P-value
Age	1.07	1.07	1.08	<0.001	1.06	1.05	1.07	<0.0001
DM	2.55	1.98	3.28	<0.001	1.57	1.11	2.23	0.011
HTN	2.28	1.93	2.70	<0.001	1.51	1.22	1.87	<0.0001
CHF	2.00	1.25	3.19	0.004	0.98	0.60	1.61	0.936
MI other	2.27	1.71	3.03	<0.001	1.36	0.96	1.93	0.088
Aortic atherosclerosis	1.21	0.39	3.75	0.746	0.70	0.22	2.22	0.548
COPD	1.55	1.24	1.95	<0.001	1.16	0.91	1.47	0.238
CKD	1.38	0.88	2.15	0.158	0.96	0.61	1.52	0.869
Thyroid disease	1.00	0.66	1.52	0.988	0.90	0.59	1.38	0.640
VHD	2.14	1.02	4.51	0.045	1.40	0.59	3.35	0.449
Liver disase	1.22	0.94	1.57	0.135	0.95	0.73	1.24	0.712
Sleep apenea	1.00	0.67	1.49	1.000	0.73	0.48	1.10	0.135
**Treatment group**								
Estradiol	1.00				1.00			
CEE	**1.50**	**1.23**	**1.82**	**<0.001**	**1.26**	**1.03**	**1.54**	**0.025**

AHR, adjusted hazards ration; CEE, conjugated equine estrogens; CHR, crude hazards ratio; CHF, congestive heart failure; CI, confidence interval; CKD, chronic kidney diease; COPD, chronic obstructive pulmonary disease; DM, diabetes mellitus; MACE, major adverse cardiac events; MI, myocardial infarction; VHD, valvular heart disease.
