# Supplementary material for: Hormone replacement therapy and risk of atrial fibrillation in Taiwanese menopause women: A nationwide cohort study
Source: Sci Rep. 2016 Apr 7;6:24132. doi: 10.1038/srep24132 (PMC4823790; doi:10.1038/srep24132)
Supplement: Supplementary Information [file srep24132-s1.doc]

**Supplementary Materials**

**Hormone replacement therapy and risk of atrial fibrillation in Taiwanese menopause women: A nationwide cohort study**

Wei–Chung Tsai, Yaw-Bin Haung, Hsuan-Fu Kuo, Wei-Hua Tang, Po–Chao Hsu, Ho–Ming Su, Tsung–Hsien Lin, Chih–Sheng Chu, Shih-Jie [Jhuo](mailto:Jhuo：siegfred_tw@yahoo.com.tw), Kun–Tai Lee, Sheng–Hsiung Sheu, Chung-Yu Chen, Ming–Tsang Wu, Wen–Ter Lai

**eTable 1. Clinical summary between Estradiol and CEE group after propensity-score-matched strategy**.

| Variable | Total (n=3453) | | Estradiol (n=1151) | | CEE (n=2302) | |  |
| --- | --- | --- | --- | --- | --- | --- | --- |
|  | No | % | No | % | No | % | p |
| **Age** (year±SD) | 55.06 (±7.56) | | 55.04 (±7.69) | | 55.08 (±7.42) | | 0.877 |
| **Income^a^** |  |  |  |  |  |  |  |
| High | 704 | 20.39 | 215 | 18.68 | 489 | 21.24 | 0.305 |
| Low | 2749 | 79.61 | 936 | 81.32 | 1813 | 78.76 |  |
| **Duration** |  |  |  |  |  |  |  |
| Total follow up (year±SD) | 7.64 (±2.98) | | 6.65 (±3.01) | | 8.27 (±2.96) | | 0.384 |
| Drug exposure (year±SD) | 0.70 (±1.04) | | 0.50 (±0.85) | | 0.76 (±1.15) | | 0.210 |
| **Co-morbidities** |  | |  | |  | |  |
| Diabetes | 148 | 4.29 | 51 | 4.43 | 97 | 4.21 | 0.766 |
| Hypertension | 675 | 19.55 | 228 | 19.81 | 447 | 19.42 | 0.785 |
| CHF | 41 | 1.19 | 14 | 1.22 | 27 | 1.17 | 0.912 |
| MI other | 108 | 3.13 | 36 | 3.13 | 72 | 3.13 | 1.000 |
| Aortic atherosclerosis | 15 | 0.43 | 5 | 0.43 | 10 | 0.43 | 1.000 |
| COPD | 287 | 8.31 | 99 | 8.60 | 188 | 8.17 | 0.663 |
| CKD | 61 | 1.77 | 20 | 1.74 | 41 | 1.78 | 0.927 |
| Thyroid disease | 120 | 3.48 | 41 | 3.56 | 79 | 3.43 | 0.844 |
| VHD | 17 | 0.49 | 4 | 0.35 | 13 | 0.56 | 0.390 |
| Liver disase | 305 | 8.83 | 107 | 9.30 | 198 | 8.60 | 0.500 |
| Sleep apenea | 187 | 5.42 | 74 | 6.43 | 113 | 4.91 | 0.060 |
| **Prescribed Drugs** |  |  |  |  |  |  |  |
| ACEI | 230 | 6.66 | 86 | 7.47 | 144 | 6.26 | 0.177 |
| ARB | 59 | 1.71 | 25 | 2.17 | 34 | 1.48 | 0.137 |
| BB | 255 | 7.38 | 85 | 7.38 | 170 | 7.38 | 1.000 |
| CCB | 76 | 2.20 | 21 | 1.82 | 55 | 2.39 | 0.286 |
| Diuretics | 259 | 7.50 | 100 | 8.69 | 159 | 6.91 | 0.061 |
| Nitrate | 61 | 1.77 | 19 | 1.65 | 42 | 1.82 | 0.715 |
| Statin | 106 | 3.07 | 55 | 4.78 | 51 | 2.22 | <0.001 |
| Aspirin | 82 | 2.37 | 29 | 2.52 | 53 | 2.30 | 0.693 |
| Warfarin | 6 | 0.17 | 1 | 0.09 | 5 | 0.22 | 0.386 |
| Steroid | 1031 | 29.86 | 365 | 31.71 | 666 | 28.93 | 0.092 |
| NSAIDs | 2144 | 62.09 | 762 | 66.20 | 1382 | 60.00 | <0.001 |
| Cox-II inhibitors | 29 | 0.84 | 17 | 1.48 | 12 | 0.52 | 0.004 |
| OAD | 208 | 6.02 | 82 | 7.12 | 126 | 5.47 | 0.055 |
| Alpha-blocker | 29 | 0.84 | 11 | 0.96 | 18 | 0.78 | 0.598 |
| Amiodarone | 0 | 0.00 | 0 | - | 0 | - | - |
| ACEI, angiotensin-converting enzyme inhibitors; ARB, angiotensin receptor blockers; BB, beta-blockers; CCB, calcium channel blockers; CHF, congestive heart failure; CKD, chronic kidney disease; COPD, chronic obstructive pulmonary disease; MI, myocardial infarction; OAD, oral Anti-diabetic agent; VHD, valvular heart disease; NSAIDs, non-steroidal anti-inflammatory drugs.  ^a^ Individual yearly gross income over NT$894,574 defined as high. The national average of annual household income in 2005 was around NT$894,574. (Source: Directorate General of Budget, Accounting and Statistics, Executive Yuan. Report on the Survey of Family Income and Expenditure in Taiwan Area of Republic | | | | | | | |

| **eTable 2. Cox proportional hazards model analysis for AF after propensity-score-matched strategy.** | | | | | | | | | | |
| --- | --- | --- | --- | --- | --- | --- | --- | --- | --- | --- |
| Variable |  | CHR | lower 95%CI | upper 95%CI | P-value |  | AHR | lower 95%CI | upper 95%CI | P-value |
| Age |  | 1.10 | 1.07 | 1.13 | <0.001 |  | 1.09 | 1.06 | 1.12 | <0.001 |
| DM |  | 1.71 | 0.62 | 4.75 | 0.103 |  | 2.23 | 0.60 | 8.31 | 0.233 |
| hypertension |  | 3.04 | 1.76 | 5.26 | <0.001 |  | 1.10 | 0.53 | 2.29 | 0.789 |
| CHF |  | 1.55 | 0.22 | 11.23 | 0.663 |  | 0.44 | 0.06 | 3.48 | 0.437 |
| MI |  | 5.22 | 2.35 | 11.57 | <0.001 |  | 1.21 | 0.44 | 3.33 | 0.718 |
| Aortic atherosclerosis |  | - | - | - | - |  | - | - | - | - |
| COPD |  | 1.60 | 0.72 | 3.53 | 0.250 |  | 1.08 | 0.46 | 2.52 | 0.859 |
| CKD |  | 2.92 | 0.91 | 9.37 | 0.072 |  | 1.92 | 0.56 | 6.57 | 0.297 |
| Thyroid disease |  | 2.38 | 0.86 | 6.58 | 0.096 |  | 2.49 | 0.85 | 7.31 | 0.095 |
| VHD |  | 4.72 | 0.65 | 34.15 | 0.125 |  | 2.79 | 0.35 | 22.36 | 0.335 |
| Liver disase |  | 0.82 | 0.30 | 2.28 | 0.709 |  | 0.62 | 0.21 | 1.80 | 0.377 |
| Sleep apenea |  | 1.29 | 0.40 | 4.15 | 0.668 |  | 0.59 | 0.17 | 2.07 | 0.414 |
| **Treatment group** |  |  |  |  |  |  |  |  |  |  |
| **Estradiol** |  | **1.00** |  |  |  |  | **1.00** |  |  |  |
| **CEE** |  | **2.06** | **1.01** | **4.23** | **0.048** |  | **2.17** | **1.05** | **4.47** | **0.036** |
| AF, atrial fibrillation; AHR, adjusted hazards ration; CEE, conjugated equine estrogens; CHR, crude hazards ratio; CHF, congestive heart failure; CI, confidence interval; CKD, chronic kidney diease; COPD, chronic obstructive pulmonary disease; DM, diabetes mellitus; MI, myocardial infarction; VHD, valvular heart disease. | | | | | | | | | | |

| **eTable 3. Cox proportional hazards model analysis for stroke after propensity-score- matched strategy.** | | | | | | | | | | |
| --- | --- | --- | --- | --- | --- | --- | --- | --- | --- | --- |
| Variable |  | CHR | lower 95%CI | upper 95%CI | P-value |  | AHR | lower 95%CI | upper 95%CI | P-value |
| Age |  | 1.07 | 1.05 | 1.08 | <0.001 |  | 1.06 | 1.04 | 1.07 | <0.001 |
| DM |  | 2.79 | 1.96 | 3.96 | <0.001 |  | 1.59 | 0.96 | 2.64 | 0.071 |
| HTN |  | 2.14 | 1.68 | 2.71 | <0.001 |  | 1.48 | 1.09 | 2.00 | 0.011 |
| CHF |  | 1.37 | 0.57 | 3.32 | 0.480 |  | 0.84 | 0.33 | 2.12 | 0.704 |
| MI other |  | 2.17 | 1.36 | 3.45 | 0.001 |  | 1.36 | 0.80 | 2.33 | 0.259 |
| Aortic atherosclerosis |  | 1.47 | 0.37 | 5.90 | 0.586 |  | 0.81 | 0.19 | 3.37 | 0.770 |
| COPD |  | 1.74 | 1.30 | 2.34 | <0.001 |  | 1.28 | 0.99 | 1.64 | 0.055 |
| CKD |  | 0.89 | 0.37 | 2.15 | 0.794 |  | 0.77 | 0.31 | 1.88 | 0.563 |
| Thyroid disease |  | 1.15 | 0.66 | 2.00 | 0.630 |  | 1.06 | 0.60 | 1.87 | 0.845 |
| VHD |  | 1.65 | 0.41 | 6.61 | 0.483 |  | 1.68 | 0.39 | 7.26 | 0.484 |
| Liver disase |  | 1.08 | 0.74 | 1.58 | 0.679 |  | 0.93 | 0.63 | 1.36 | 0.692 |
| Sleep apenea |  | 2.05 | 1.24 | 3.40 | 0.005 |  | 1.33 | 0.78 | 2.26 | 0.300 |
| **Treatment group** |  |  |  |  |  |  |  |  |  |  |
| **Estradiol** |  | **1.00** |  |  |  |  | **1.00** |  |  |  |
| **CEE** |  | **1.62** | **1.34** | **1.92** | **<0.001** |  | **1.33** | **1.02** | **1.73** | **0.036** |
| AHR, adjusted hazards ration; CEE, conjugated equine estrogens; CHR, crude hazards ratio; CHF, congestive heart failure; CI, confidence interval; CKD, chronic kidney diease; COPD, chronic obstructive pulmonary disease; DM, diabetes mellitus; MI, myocardial infarction; VHD, valvular heart disease. | | | | | | | | | | |

| **eTable 4. Cox proportional hazards model analysis for MACE after propensity-score-matched strategy.** | | | | | | | | | | |
| --- | --- | --- | --- | --- | --- | --- | --- | --- | --- | --- |
| Variable |  | CHR | lower 95%CI | upper 95%CI | P-value |  | AHR | lower 95%CI | upper 95%CI | P-value |
| Age |  | 1.07 | 1.06 | 1.08 | <0.001 |  | 1.06 | 1.05 | 1.08 | <0.001 |
| DM |  | 2.67 | 1.93 | 3.71 | <0.001 |  | 1.63 | 1.03 | 2.58 | 0.038 |
| HTN |  | 2.18 | 1.75 | 2.71 | <0.001 |  | 1.55 | 1.18 | 2.04 | 0.002 |
| CHF |  | 1.40 | 0.63 | 3.14 | 0.410 |  | 0.82 | 0.35 | 1.91 | 0.643 |
| MI other |  | 2.55 | 1.71 | 3.80 | <0.001 |  | 1.47 | 0.92 | 2.34 | 0.107 |
| Aortic atherosclerosis |  | 1.21 | 0.30 | 4.87 | 0.785 |  | 0.68 | 0.16 | 2.82 | 0.590 |
| COPD |  | 1.74 | 1.33 | 2.28 | <0.001 |  | 1.56 | 1.18 | 2.06 | 0.002 |
| CKD |  | 1.35 | 0.70 | 2.62 | 0.372 |  | 1.19 | 0.61 | 2.34 | 0.608 |
| Thyroid disease |  | 1.19 | 0.72 | 1.96 | 0.497 |  | 1.08 | 0.65 | 1.81 | 0.764 |
| VHD |  | 1.36 | 0.34 | 5.47 | 0.661 |  | 1.22 | 0.29 | 5.19 | 0.792 |
| Liver disase |  | 1.14 | 0.81 | 1.59 | 0.466 |  | 0.94 | 0.66 | 1.34 | 0.736 |
| Sleep apenea |  | 1.92 | 1.20 | 3.09 | 0.007 |  | 1.18 | 0.72 | 1.95 | 0.514 |
| **Treatment group** |  |  |  |  |  |  |  |  |  |  |
| **Estradiol** |  | **1.00** |  |  |  |  | **1.00** |  |  |  |
| **CEE** |  | **1.64** | **1.35** | **1.98** | **<0.001** |  | **1.33** | **1.05** | **1.69** | **0.020** |
| AHR, adjusted hazards ration; CEE, conjugated equine estrogens; CHR, crude hazards ratio; CHF, congestive heart failure; CI, confidence interval; CKD, chronic kidney diease; COPD, chronic obstructive pulmonary disease; DM, diabetes mellitus; MACE, major adverse cardiac events; MI, myocardial infarction; VHD, valvular heart disease. | | | | | | | | | | |
